# Supplementary material for: TLR9 gene polymorphism -1237T/C (rs5743836) is associated with low IgG antibody response against PvCSP variants in symptomatic P. vivax infections in Venezuela
Source: PLoS Negl Trop Dis. 2025 Jun 30;19(6):e0013262. doi: 10.1371/journal.pntd.0013262 (PMC12233907; doi:10.1371/journal.pntd.0013262)
Supplement: S1 Table — (DOCX) [file pntd.0013262.s001.docx]

**S1 Table.** Peptide sequences corresponding to the three variants (VK247, VK210, and V-like) of the *Pv*CSP repetitive region

| **Variants** | **Sequences** | **Theoretical MW (g/mol)** |
| --- | --- | --- |
| VK247 | Ac-ANGAGNQPGANGAGNQPGANGAGNQPGANGAGN-NH2 | 2,843.8 |
| VK210 | Ac-GNAAGNAAGNDAGNAAGNAAGNAAGNAA-NH2 | 2,293.3 |
| V-like | Ac-APGANQEGGAAAPGANQEGGAAAPGANQEGGAA-NH2 | 2,830.8 |

MW: molecular weight
