# Supplementary material for: TLR9 gene polymorphism -1237T/C (rs5743836) is associated with low IgG antibody response against PvCSP variants in symptomatic P. vivax infections in Venezuela
Source: PLoS Negl Trop Dis. 2025 Jun 30;19(6):e0013262. doi: 10.1371/journal.pntd.0013262 (PMC12233907; doi:10.1371/journal.pntd.0013262)
Supplement: S2 Table — (DOCX) [file pntd.0013262.s002.docx]

**S2 Table.** Description of polymorphisms, primer sequences, PCR amplification program, restriction enzymes, and fragments resulting from the genotyping of *TLR9* gene SNPs

| **Polymorphisms** | **Primer sequences** | **PCR amplification program** | **Restriction enzymes** | **Fragments resulting (bp)** |
| --- | --- | --- | --- | --- |
| **rs5743836 (-1237T/C)** | P: 5’-TCATTCAGCCTTCACTCAGA-3’  Q: 5’-CACATTCAGCCCCTAGAGGG-3’  W: 5’-GGCGGCGGGGGTGCTGTTCCCTCTGCCTGA-3’  M: 5’-GGGCCGGGGGATGAGACTTGGGGGAGTTTC-3’ | 95 °C for 5 min, 35 × (95 °C for 30 sec, 65 °C for 30 sec, 72 °C for 30 sec), 72 °C for 10 min | NA | T/T: 644, 395  T/C: 644, 395, 275  C/C: 644, 275 |
| **rs352140 (1635A/G)** | Fwd: 5’-CAGCTCGGCATCTTCAGGGCCTTC-3’  Rev: 5’-CAGTGCATTGCCGCTGAAGTCCAG-3’ | 95 °C for 5 min, 35 × (95 °C for 30 sec, 65 °C for 30 sec, 72 °C for 30 sec), 72 °C for 10 min | BstUI | A/A: 612  A/G: 612, 417, 195  G/G: 417, 195 |
| **rs187084 (1486C/T)** | Fwd: 5’-CTATGGAGCCTGCCTGCCATGATACC-3’  Rev: 5’-CTGGTCACATTCAGCCCCTAGAG-3’ | 95 °C for 5 min, 35 × (95 °C for 30 sec, 62 °C for 30 sec, 72 °C for 30 sec), 72 °C for 10 min | AflII | C/C: 755  C/T: 755, 505, 250  T/T: 505, 250 |

rs: reference sequence, Fwd: forward, Rev: reverse, NA: not applicable, bp: base pairs
