# Supplementary material for: TLR9 gene polymorphism -1237T/C (rs5743836) is associated with low IgG antibody response against PvCSP variants in symptomatic P. vivax infections in Venezuela
Source: PLoS Negl Trop Dis. 2025 Jun 30;19(6):e0013262. doi: 10.1371/journal.pntd.0013262 (PMC12233907; doi:10.1371/journal.pntd.0013262)
Supplement: S3 Table — (DOCX) [file pntd.0013262.s003.docx]

**S3 Table.** Clinical-epidemiological characteristics of individuals infected with *P. vivax* by their IgG antibody response level against the VK247 *Pv*CSP variant

| **Clinical-epidemiological characteristics** | **Responder against the VK247 *Pv*CSP variant** | | | ***p* value** |
| --- | --- | --- | --- | --- |
|  | **Low (*n* = 85, 42.9%)** | **Medium (*n* = 95, 48%)** | **High (*n* = 18, 9.1%)** |  |
| Age, median (IQR), years | 28 (20-42) | 31 (21-45) | 28 (20-39) | 0.605^*^ |
| Sex, *n* (%) |  |  |  | 0.381^†^ |
| Male | 47 (55.3) | 58 (61.1) | 13 (72.2) |  |
| Female | 38 (44.7) | 37 (38.9) | 5 (27.8) |  |
| Education level, *n* (%) |  |  |  | 0.376^‡^ |
| None | 0 (0) | 3 (3.2) | 0 (0) |  |
| Primary school | 33 (38.8) | 34 (35.8) | 7 (38.9) |  |
| High school | 36 (42.4) | 46 (48.4) | 6 (33.3) |  |
| College | 16 (18.8) | 12 (12.6) | 5 (27.8) |  |
| Occupation, *n* (%) |  |  |  | 0.919^‡^ |
| Illegal gold mining | 52 (61.2) | 46 (48.4) | 12 (66.7) |  |
| Homemaker | 12 (14.1) | 13 (13.7) | 3 (16.7) |  |
| Farmer | 5 (5.9) | 9 (9.5) | 1 (5.6) |  |
| Government employee | 4 (4.7) | 4 (4.2) | 1 (5.6) |  |
| Student | 2 (2.4) | 6 (6.3) | 0 (0) |  |
| Worker | 2 (2.4) | 6 (6.3) | 0 (0) |  |
| Teacher | 3 (3.5) | 3 (3.2) | 1 (5.6) |  |
| Merchant | 1 (1.2) | 3 (3.2) | 0 (0) |  |
| Other | 4 (4.7) | 5 (5.3) | 0 (0) |  |
| PAI (municipality), *n* (%) |  |  |  | 0.989^‡^ |
| Sifontes | 33 (38.8) | 33 (34.7) | 8 (44.4) |  |
| Sucre | 25 (29.4) | 23 (24.2) | 4 (22.2) |  |
| Angostura del Orinoco | 16 (18.8) | 25 (26.3) | 5 (27.8) |  |
| Angostura | 3 (3.5) | 5 (5.3) | 0 (0) |  |
| Piar | 3 (3.5) | 4 (4.2) | 1 (5.6) |  |
| El Callao | 2 (2.4) | 2 (2.1) | 0 (0) |  |
| Caroní | 2 (2.4) | 2 (2.1) | 0 (0) |  |
| Gran Sabana | 1 (1.2) | 0 (0) | 0 (0) |  |
| Cedeño | 0 (0) | 1 (1.1) | 0 (0) |  |
| Parasitemia, median (IQR), /µL | 4,300 (3,500-5,600) | 4,200 (3,200-5,700) | 4,600 (4,100-6,700) | 0.366^*^ |
| Parasitemia, *n* (%) |  |  |  | 0.857^†^ |
| Low | 53 (62.4) | 59 (62.1) | 10 (55.6) |  |
| High | 32 (37.6) | 36 (37.9) | 8 (44.4) |  |
| Previous malaria, *n* (%) |  |  |  | 0.286^†^ |
| No | 18 (21.2) | 20 (21.1) | 1 (5.6) |  |
| Yes | 67 (78.8) | 75 (78.9) | 17 (94.4) |  |
| No. of total episodes, median (IQR) | 6 (2-13) | 5 (2-11) | 8 (4-18) | 0.44^*^ |
| No. of episodes in the last year, median (IQR) | 2 (1-5) | 2 (1-4) | 2 (1-6) | 0.311^*^ |
| Days since last episode, median (IQR) | 86 (57-122) | 95 (59-137) | 80 (69-109) | 0.818^*^ |

*Kruskal-Wallis test, †Pearson’s chi-square test, ‡Fisher’s exact test. IQR: interquartile range. PAI: probable area of infection
