# Supplementary material for: TLR9 gene polymorphism -1237T/C (rs5743836) is associated with low IgG antibody response against PvCSP variants in symptomatic P. vivax infections in Venezuela
Source: PLoS Negl Trop Dis. 2025 Jun 30;19(6):e0013262. doi: 10.1371/journal.pntd.0013262 (PMC12233907; doi:10.1371/journal.pntd.0013262)
Supplement: S4 Table — (DOCX) [file pntd.0013262.s004.docx]

**S4 Table.** Clinical-epidemiological characteristics of individuals infected with *P. vivax* by their IgG antibody response level against the VK210 *Pv*CSP variant

| **Clinical-epidemiological characteristics** | **Responder against the VK210 *Pv*CSP variant** | | | ***p* value** |
| --- | --- | --- | --- | --- |
|  | **Low (*n* = 127, 63.5%)** | **Medium (*n* = 61, 30.5%)** | **High (*n* = 12, 6%)** |  |
| Age, median (IQR), years | 29 (20-43) | 29 (19-44) | 29 (25-50) | 0.678^*^ |
| Sex, *n* (%) |  |  |  | 0.831^†^ |
| Male | 74 (58.3) | 37 (60.7) | 8 (66.7) |  |
| Female | 53 (41.7) | 24 (39.3) | 4 (33.3) |  |
| Education level, *n* (%) |  |  |  | 0.146^‡^ |
| None | 1 (0.8) | 2 (3.3) | 0 (0) |  |
| Primary school | 46 (36.2) | 24 (39.3) | 4 (33.3) |  |
| High school | 56 (44.1) | 29 (47.5) | 3 (25) |  |
| College | 24 (18.9) | 6 (9.8) | 5 (41.7) |  |
| Occupation, *n* (%) |  |  |  | 0.795^‡^ |
| Illegal gold mining | 70 (55.1) | 35 (57.4) | 6 (50) |  |
| Homemaker | 17 (13.4) | 9 (14.8) | 2 (16.7) |  |
| Farmer | 9 (7.1) | 3 (4.9) | 2 (16.7) |  |
| Government employee | 7 (5.5) | 2 (3.3) | 0 (0) |  |
| Student | 4 (3.1) | 4 (6.6) | 0 (0) |  |
| Worker | 4 (3.1) | 4 (6.6) | 0 (0) |  |
| Teacher | 5 (3.9) | 1 (1.6) | 1 (8.3) |  |
| Merchant | 3 (2.4) | 2 (3.3) | 0 (0) |  |
| Other | 8 (6.3) | 1 (1.6) | 1 (8.3) |  |
| PAI (municipality), *n* (%) |  |  |  | 0.252^‡^ |
| Sifontes | 44 (34.6) | 23 (37.7) | 7 (58.3) |  |
| Sucre | 38 (29.9) | 16 (26.2) | 0 (0) |  |
| Angostura del Orinoco | 26 (20.5) | 18 (29.5) | 2 (16.7) |  |
| Angostura | 5 (3.9) | 2 (3.3) | 1 (8.3) |  |
| Piar | 6 (4.7) | 1 (1.6) | 1 (8.3) |  |
| El Callao | 2 (1.6) | 1 (1.6) | 1 (8.3) |  |
| Caroní | 4 (3.1) | 0 (0) | 0 (0) |  |
| Gran Sabana | 1 (0.8) | 0 (0) | 0 (0) |  |
| Cedeño | 1 (0.8) | 0 (0) | 0 (0) |  |
| Parasitemia, median (IQR), /µL | 4,300 (3,500-5,600) | 4,200 (3,200-6,100) | 4,300 (4,150-5,400) | 0.718^*^ |
| Parasitemia, *n* (%) |  |  |  | 0.857^‡^ |
| Low | 77 (60.6) | 39 (63.9) | 8 (66.7) |  |
| High | 50 (39.4) | 22 (36.1) | 4 (33.3) |  |
| Previous malaria, *n* (%) |  |  |  | 0.139^†^ |
| No | 24 (18.9) | 15 (24.6) | 0 (0) |  |
| Yes | 103 (81.1) | 46 (75.4) | 12 (100) |  |
| No. of total episodes, median (IQR) | 5 (2-12) | 5 (2-10) | 6.5 (3-15) | 0.856^*^ |
| No. of episodes in the last year, median (IQR) | 2 (1-5) | 2 (1-5) | 2 (1-5.5) | 0.646^*^ |
| Days since last episode, median (IQR) | 95 (57-133) | 79 (60-129) | 87 (68.5-105) | 0.832^*^ |

*Kruskal-Wallis test, †Pearson’s chi-square test, ‡Fisher’s exact test. IQR: interquartile range. PAI: probable area of infection
