# Supplementary material for: TLR9 gene polymorphism -1237T/C (rs5743836) is associated with low IgG antibody response against PvCSP variants in symptomatic P. vivax infections in Venezuela
Source: PLoS Negl Trop Dis. 2025 Jun 30;19(6):e0013262. doi: 10.1371/journal.pntd.0013262 (PMC12233907; doi:10.1371/journal.pntd.0013262)
Supplement: S5 Table — (DOCX) [file pntd.0013262.s005.docx]

**S5 Table.** Clinical-epidemiological characteristics of individuals infected with *P. vivax* by their IgG antibody response level against the V-like *Pv*CSP variant

| **Clinical-epidemiological characteristics** | **Responder against the V-like *Pv*CSP variant** | | | ***p* value** |
| --- | --- | --- | --- | --- |
|  | **Low (*n* = 137, 67.2%)** | **Medium (*n* = 52, 25.5%)** | **High (*n* = 15, 7.4%)** |  |
| Age, median (IQR), years | 28 (21-42) | 30 (20-46) | 30 (22-47) | 0.602^*^ |
| Sex, *n* (%) |  |  |  | 0.79^†^ |
| Male | 80 (58.4) | 32 (61.5) | 10 (66.7) |  |
| Female | 57 (41.6) | 20 (38.5) | 5 (33.3) |  |
| Education level, *n* (%) |  |  |  | 0.536^‡^ |
| None | 3 (2.2) | 0 (0) | 0 (0) |  |
| Primary school | 48 (35) | 21 (40.4) | 5 (33.3) |  |
| High school | 62 (45.3) | 25 (48.1) | 5 (33.3) |  |
| College | 24 (17.5) | 6 (11.5) | 5 (33.3) |  |
| Occupation, *n* (%) |  |  |  | 0.718^‡^ |
| Illegal gold mining | 78 (56.9) | 29 (55.8) | 8 (53.3) |  |
| Homemaker | 18 (13.1) | 7 (13.5) | 3 (20) |  |
| Farmer | 11 (8) | 2 (3.8) | 2 (13.3) |  |
| Government employee | 7 (5.1) | 2 (3.8) | 0 (0) |  |
| Student | 4 (2.9) | 4 (7.7) | 0 (0) |  |
| Worker | 4 (2.9) | 4 (7.7) | 0 (0) |  |
| Teacher | 5 (3.6) | 1 (1.9) | 1 (6.7) |  |
| Merchant | 2 (1.5) | 2 (3.8) | 0 (0) |  |
| Other | 8 (5.8) | 1 (1.9) | 1 (6.7) |  |
| PAI (municipality), *n* (%) |  |  |  | 0.689^‡^ |
| Sifontes | 48 (35) | 21 (40.4) | 8 (53.3) |  |
| Sucre | 40 (29.2) | 14 (26.9) | 1 (6.7) |  |
| Angostura del Orinoco | 30 (21.9) | 13 (25) | 3 (20) |  |
| Angostura | 5 (3.6) | 2 (3.8) | 1 (6.7) |  |
| Piar | 6 (4.4) | 1 (1.9) | 1 (6.7) |  |
| El Callao | 2 (1.5) | 1 (1.9) | 1 (6.7) |  |
| Caroní | 4 (2.9) | 0 (0) | 0 (0) |  |
| Gran Sabana | 1 (0.7) | 0 (0) | 0 (0) |  |
| Cedeño | 1 (0.7) | 0 (0) | 0 (0) |  |
| Parasitemia, median (IQR), /µL | 4,300 (3,500-5,700) | 4,150 (3,200-5,900) | 4,300 (4,100-5,650) | 0.665^*^ |
| Parasitemia, *n* (%) |  |  |  | 0.823^†^ |
| Low | 83 (60.6) | 34 (65.4) | 9 (60) |  |
| High | 54 (39.4) | 18 (34.6) | 6 (40) |  |
| Previous malaria, *n* (%) |  |  |  | 0.129^†^ |
| No | 27 (19.7) | 12 (23.1) | 0 (0) |  |
| Yes | 110 (80.3) | 40 (76.9) | 15 (100) |  |
| No. of total episodes, median (IQR) | 5 (2-11) | 6 (2-11.5) | 9 (3-18) | 0.514^*^ |
| No. of episodes in the last year, median (IQR) | 2 (1-5) | 2 (1-5) | 2 (1-7) | 0.359^*^ |
| Days since last episode, median (IQR) | 94.5 (57-113) | 80 (58-134) | 86 (69-109) | 0.975^*^ |

*Kruskal-Wallis test, †Pearson’s chi-square test, ‡Fisher’s exact test. IQR: interquartile range. PAI: probable area of infection
