# Supplementary material for: TLR9 gene polymorphism -1237T/C (rs5743836) is associated with low IgG antibody response against PvCSP variants in symptomatic P. vivax infections in Venezuela
Source: PLoS Negl Trop Dis. 2025 Jun 30;19(6):e0013262. doi: 10.1371/journal.pntd.0013262 (PMC12233907; doi:10.1371/journal.pntd.0013262)
Supplement: S6 Table — (DOCX) [file pntd.0013262.s006.docx]

**S6 Table.** Clinical-epidemiological characteristics of individuals infected with *P. vivax* by their IgG antibody response level against *Pv*MSP-1_19_

| **Clinical-epidemiological characteristics** | **Responder against *Pv*MSP-1_19_** | | | ***p* value** |
| --- | --- | --- | --- | --- |
|  | **Low (*n* = 47, 22.6%)** | **Medium (*n* = 63, 30.3%)** | **High (*n* = 98, 47.1%)** |  |
| Age, median (IQR), years | 27 (19-43) | 33 (22-43) | 28 (20-42) | 0.498^*^ |
| Sex, *n* (%) |  |  |  | 0.657^†^ |
| Male | 31 (66) | 39 (61.9) | 57 (58.2) |  |
| Female | 16 (34) | 24 (38.1) | 41 (41.8) |  |
| Education level, *n* (%) |  |  |  | 0.163^†^ |
| None | 0 (0) | 0 (0) | 3 (3.1) |  |
| Primary school | 23 (48.9) | 21 (33.3) | 33 (33.7) |  |
| High school | 19 (40.4) | 27 (42.9) | 47 (48) |  |
| College | 5 (10.6) | 15 (23.8) | 15 (15.3) |  |
| Occupation, *n* (%) |  |  |  | 0.819^‡^ |
| Illegal gold mining | 28 (59.6) | 29 (46) | 59 (60.2) |  |
| Homemaker | 5 (10.6) | 11 (17.5) | 12 (12.2) |  |
| Farmer | 4 (8.5) | 5 (7.9) | 6 (6.1) |  |
| Government employee | 2 (4.3) | 4 (6.3) | 3 (3.1) |  |
| Student | 3 (6.4) | 1 (1.6) | 5 (5.1) |  |
| Worker | 2 (4.3) | 5 (7.9) | 2 (2) |  |
| Teacher | 1 (2.1) | 3 (4.8) | 3 (3.1) |  |
| Merchant | 1 (2.1) | 2 (3.2) | 2 (2) |  |
| Other | 1 (2.1) | 3 (4.8) | 6 (6.1) |  |
| PAI (municipality), *n* (%) |  |  |  | 0.956^‡^ |
| Sifontes | 18 (38.3) | 25 (39.7) | 36 (36.7) |  |
| Sucre | 11 (23.4) | 16 (25.4) | 28 (28.6) |  |
| Angostura del Orinoco | 14 (29.8) | 15 (23.8) | 18 (18.4) |  |
| Angostura | 1 (2.1) | 3 (4.8) | 5 (5.1) |  |
| Piar | 1 (2.1) | 2 (3.2) | 5 (5.1) |  |
| El Callao | 1 (2.1) | 1 (1.6) | 2 (2) |  |
| Caroní | 1 (2.1) | 0 (0) | 3 (3.1) |  |
| Gran Sabana | 0 (0) | 1 (1.6) | 0 (0) |  |
| Cedeño | 0 (0) | 0 (0) | 1 (1) |  |
| Parasitemia, median (IQR), /µL | 4,500 (3,500-6,300) | 4,300 (3,450-5,700) | 4,275 (3,600-5,900) | 0.947^*^ |
| Parasitemia, *n* (%) |  |  |  | 0.452^†^ |
| Low | 28 (59.6) | 35 (55.6) | 64 (65.3) |  |
| High | 19 (40.4) | 28 (44.4) | 34 (34.7) |  |
| Previous malaria, *n* (%) |  |  |  | 0.038^†^ |
| No | 15 (31.9) | 11 (17.5) | 14 (14.3) |  |
| Yes | 32 (68.1) | 52 (82.5) | 84 (85.7) |  |
| No. of total episodes, median (IQR) | 6.5 (2.5-12.5) | 5 (2-10.5) | 5 (2-14) | 0.268^*^ |
| No. of episodes in the last year, median (IQR) | 2 (1-5) | 2 (1-5) | 2 (1-4) | 0.791^*^ |
| Days since last episode, median (IQR) | 87.5 (58-131) | 79.5 (56-116) | 95 (58-157) | 0.408^*^ |

*Kruskal-Wallis test, †Pearson’s chi-square test, ‡Fisher’s exact test. IQR: interquartile range. PAI: probable area of infection
