# Supplementary material for: TLR9 gene polymorphism -1237T/C (rs5743836) is associated with low IgG antibody response against PvCSP variants in symptomatic P. vivax infections in Venezuela
Source: PLoS Negl Trop Dis. 2025 Jun 30;19(6):e0013262. doi: 10.1371/journal.pntd.0013262 (PMC12233907; doi:10.1371/journal.pntd.0013262)
Supplement: S7 Table — (DOCX) [file pntd.0013262.s007.docx]

**S7 Table.** Association of genotypic frequencies of *TLR9* gene SNPs with IgG antibody response level against the VK210 *Pv*CSP variant

| **SNPs** | **Inheritance models** | **Genotypes** | **Responder against the VK210 *Pv*CSP variant** | | **OR^*^ (95% CI)** | ***p* value** | **AIC** |
| --- | --- | --- | --- | --- | --- | --- | --- |
|  |  |  | **Low (*n* = 182, 91%)** | **High (*n* = 18, 9%)** |  |  |  |
| rs5743836 | Codominant | T/T | 51 (28) | 8 (44.4) | 1 | 0.13 | 141.6 |
|  |  | T/C | 127 (69.8) | 8 (44.4) | 0.47 (0.16-1.39) |  |  |
|  |  | C/C | 4 (2.2) | 2 (11.1) | 3.09 (0.4-23.72) |  |  |
|  | Dominant | T/T | 51 (28) | 8 (44.4) | 1 | 0.28 | 142.5 |
|  |  | T/C-C/C | 131 (72) | 10 (55.6) | 0.55 (0.19-1.58) |  |  |
|  | Recessive | T/T-T/C | 178 (97.8) | 16 (88.9) | 1 | 0.13 | 141.4 |
|  |  | C/C | 4 (2.2) | 2 (11.1) | 5.04 (0.72-35.1) |  |  |
|  | Overdominant | T/T-C/C | 55 (30.2) | 10 (55.6) | 1 | 0.081 | 140.7 |
|  |  | T/C | 127 (69.8) | 8 (44.4) | 0.4 (0.14-1.12) |  |  |
|  | Additive | – | – | – | 0.84 (0.32-2.22) | 0.73 | 143.6 |
| rs352140 | Codominant | A/A | 41 (22.5) | 4 (22.2) | 1 | 0.86 | 145.4 |
|  |  | A/G | 103 (56.6) | 11 (61.1) | 0.93 (0.27-3.24) |  |  |
|  |  | G/G | 38 (20.9) | 3 (16.7) | 0.66 (0.13-3.47) |  |  |
|  | Dominant | A/A | 41 (22.5) | 4 (22.2) | 1 | 0.82 | 143.7 |
|  |  | A/G-G/G | 141 (77.5) | 14 (77.8) | 0.86 (0.26-2.9) |  |  |
|  | Recessive | A/A-A/G | 144 (79.1) | 15 (83.3) | 1 | 0.6 | 143.4 |
|  |  | G/G | 38 (20.9) | 3 (16.7) | 0.7 (0.17-2.77) |  |  |
|  | Overdominant | A/A-G/G | 79 (43.4) | 7 (38.9) | 1 | 0.82 | 143.7 |
|  |  | A/G | 103 (56.6) | 11 (61.1) | 1.12 (0.4-3.18) |  |  |
|  | Additive | – | – | – | 0.82 (0.37-1.83) | 0.64 | 143.5 |

^*^Adjusted for age, sex, mining occupation, probable area of infection, previous malaria, number of total episodes, and days since last episode. OR: odds ratio. CI: confidence interval. AIC: Akaike information criterion
