# Supplementary material for: TLR9 gene polymorphism -1237T/C (rs5743836) is associated with low IgG antibody response against PvCSP variants in symptomatic P. vivax infections in Venezuela
Source: PLoS Negl Trop Dis. 2025 Jun 30;19(6):e0013262. doi: 10.1371/journal.pntd.0013262 (PMC12233907; doi:10.1371/journal.pntd.0013262)
Supplement: S8 Table — (DOCX) [file pntd.0013262.s008.docx]

**S8 Table.** Association of genotypic frequencies of *TLR9* gene SNPs with IgG antibody response level against *Pv*MSP-1_19_

| **SNPs** | **Inheritance models** | **Genotypes** | **Responder against *Pv*MSP-1_19_** | | **OR^*^ (95% CI)** | ***p* value** | **AIC** |
| --- | --- | --- | --- | --- | --- | --- | --- |
|  |  |  | **Low (*n* = 59, 28.4%)** | **High (*n* = 149, 71.6%)** |  |  |  |
| rs5743836 | Codominant | T/T | 15 (25.4) | 48 (32.2) | 1 | 0.081 | 266.6 |
|  |  | T/C | 44 (74.6) | 95 (63.8) | 0.70 (0.35-1.43) |  |  |
|  |  | C/C | 0 (0) | 6 (4) | 4.15 (0.22-78.01) |  |  |
|  | Dominant | T/T | 15 (25.4) | 48 (32.2) | 1 | 0.41 | 268.9 |
|  |  | T/C-C/C | 44 (74.6) | 101 (67.8) | 0.75 (0.37-1.51) |  |  |
|  | Recessive | T/T-T/C | 59 (100) | 143 (96) | 1 | 0.16 | 267.7 |
|  |  | C/C | 0 (0) | 6 (4) | 5.39 (0.3-97.19) |  |  |
|  | Overdominant | T/T-C/C | 15 (25.4) | 54 (36.2) | 1 | 0.18 | 267.8 |
|  |  | T/C | 44 (74.6) | 95 (63.8) | 0.62 (0.31-1.25) |  |  |
|  | Additive | – | – | – | 0.93 (0.49-1.74) | 0.82 | 269.6 |
| rs352140 | Codominant | A/A | 10 (16.9) | 37 (24.8) | 1 | 0.56 | 270.5 |
|  |  | A/G | 36 (61) | 83 (55.7) | 0.66 (0.29-1.52) |  |  |
|  |  | G/G | 13 (22) | 29 (19.5) | 0.63 (0.23-1.7) |  |  |
|  | Dominant | A/A | 10 (16.9) | 37 (24.8) | 1 | 0.29 | 268.5 |
|  |  | A/G-G/G | 49 (83) | 112 (75.2) | 0.65 (0.29-1.46) |  |  |
|  | Recessive | A/A-A/G | 46 (78) | 120 (80.5) | 1 | 0.68 | 269.4 |
|  |  | G/G | 13 (22) | 29 (19.5) | 0.85 (0.39-1.84) |  |  |
|  | Overdominant | A/A-G/G | 23 (39) | 66 (44.3) | 1 | 0.59 | 269.3 |
|  |  | A/G | 36 (61) | 83 (55.7) | 0.84 (0.44-1.59) |  |  |
|  | Additive | – | – | – | 0.79 (0.49-1.29) | 0.35 | 268.7 |

^*^Adjusted for age, sex, mining occupation, probable area of infection, previous malaria, number of total episodes, and days since last episode. OR: odds ratio. CI: confidence interval. AIC: Akaike information criterion
