# Supplementary material for: TLR9 gene polymorphism -1237T/C (rs5743836) is associated with low IgG antibody response against PvCSP variants in symptomatic P. vivax infections in Venezuela
Source: PLoS Negl Trop Dis. 2025 Jun 30;19(6):e0013262. doi: 10.1371/journal.pntd.0013262 (PMC12233907; doi:10.1371/journal.pntd.0013262)
Supplement: S9 Table — (DOCX) [file pntd.0013262.s009.docx]

**S9 Table.** Association of haplotypic frequencies of *TLR9* gene SNPs with IgG antibody response level against the three *Pv*CSP variants and *Pv*MSP-1_19_

| **Haplotype** | **SNPs** | | | **All (*n* = 198)** | **Responder against the VK247 *Pv*CSP variant** | | **OR^*^ (95% CI)** | ***p* value^†^** |
| --- | --- | --- | --- | --- | --- | --- | --- | --- |
|  | **rs5743836** | **rs352140** | **rs187084** |  | **Low (*n* = 179)** | **High (*n* = 19)** |  |  |
| 1 | T | A | C | 0.3739 | 0.3658 | 0.4472 | 1 | – |
| 2 | C | G | T | 0.2121 | 0.2195 | 0.1531 | 0.51 (0.11-2.5) | 0.41 |
| 3 | T | G | T | 0.142 | 0.1294 | 0.2417 | 1.02 (0.19-5.6) | 0.98 |
| 4 | T | G | C | 0.1129 | 0.1193 | 0.0743 | 0.83 (0.21-3.34) | 0.79 |
| 5 | C | A | C | 0.0862 | 0.0892 | 0.0528 | 0.39 (0.04-4.15) | 0.44 |
| 6 | C | A | T | 0.055 | 0.0618 | 0 | – | 1 |
| 7 | C | G | C | 0.0179 | 0.0151 | 0.0309 | 0.18 (0.01-30.58) | 0.65 |
| **Haplotype** | **SNPs** | | | **All (*n* = 200)** | **Responder against the VK210 *Pv*CSP variant** | | **OR^*^ (95% CI)** | ***p* value^‡^** |
|  | **rs5743836** | **rs352140** | **rs187084** |  | **Low (*n* = 182)** | **High (*n* = 18)** |  |  |
| 1 | T | A | C | 0.3618 | 0.3661 | 0.2523 | 1 | – |
| 2 | C | G | T | 0.2041 | 0.2126 | 0.0578 | 0.88 (0.09-8.81) | 0.91 |
| 3 | T | G | T | 0.1478 | 0.1406 | 0.2826 | 3.79 (0.36-40) | 0.27 |
| 4 | T | G | C | 0.1229 | 0.1225 | 0.1318 | 1.14 (0.24-5.43) | 0.87 |
| 5 | C | A | C | 0.0926 | 0.0887 | 0.1992 | 3.85 (0.35-42.18) | 0.27 |
| 6 | C | A | T | 0.0556 | 0.0535 | 0.0763 | 4.28 (0.25-73.34) | 0.32 |
| 7 | C | G | C | 0.0152 | 0.0162 | 0 | – | 1 |
| **Haplotype** | **SNPs** | | | **All (*n* = 204)** | **Responder against the V-like *Pv*CSP variant** | | **OR^*^ (95% CI)** | ***p* value^§^** |
|  | **rs5743836** | **rs352140** | **rs187084** |  | **Low (*n* = 183)** | **High (*n* = 21)** |  |  |
| 1 | T | A | C | 0.3731 | 0.3718 | 0.3141 | 1 | – |
| 2 | C | G | T | 0.2102 | 0.2192 | 0.076 | 0.76 (0.14-4.02) | 0.75 |
| 3 | T | G | T | 0.1408 | 0.1327 | 0.266 | 1.84 (0.28-11.93) | 0.52 |
| 4 | T | G | C | 0.116 | 0.1185 | 0.1104 | 0.78 (0.19-3.16) | 0.73 |
| 5 | C | A | C | 0.0906 | 0.0893 | 0.1708 | 1.42 (0.2-10.29) | 0.73 |
| 6 | C | A | T | 0.0534 | 0.0525 | 0.0628 | 1.72 (0.18-16.57) | 0.64 |
| 7 | C | G | C | 0.0159 | 0.016 | 0 | – | 1 |
| **Haplotype** | **SNPs** | | | **All (*n* = 208)** | **Responder against *Pv*MSP-1_19_** | | **OR^*^ (95% CI)** | ***p* value^\|\|^** |
|  | **rs5743836** | **rs352140** | **rs187084** |  | **Low (*n* = 59)** | **High (*n* = 149)** |  |  |
| 1 | T | A | C | 0.3707 | 0.1408 | 0.3792 | 1 | – |
| 2 | C | G | T | 0.2068 | 0 | 0.1861 | – | 1 |
| 3 | T | G | T | 0.1464 | 0.3862 | 0.1572 | 0.97 (0.23-4.07) | 0.96 |
| 4 | T | G | C | 0.1199 | 0.0796 | 0.1046 | 0.68 (0.27-1.72) | 0.42 |
| 5 | C | A | C | 0.0882 | 0.3132 | 0.0849 | 0.57 (0.12-2.8) | 0.49 |
| 6 | C | A | T | 0.0531 | 0 | 0.0628 | – | 1 |
| 7 | C | G | C | 0.0149 | 0.0596 | 0.0253 | 0.74 (0.24-2.3) | 0.61 |

^*^Adjusted for age, sex, mining occupation, probable area of infection, previous malaria, number of total episodes, and days since last episode. OR: odds ratio. CI: confidence interval. *p* value of overall haplotype association = ^†^0.42, ^‡^0.81, ^§^0.84, ^||^0.54
