# Supplementary material for: TLR9 gene polymorphism -1237T/C (rs5743836) is associated with low IgG antibody response against PvCSP variants in symptomatic P. vivax infections in Venezuela
Source: PLoS Negl Trop Dis. 2025 Jun 30;19(6):e0013262. doi: 10.1371/journal.pntd.0013262 (PMC12233907; doi:10.1371/journal.pntd.0013262)
Supplement: Attachment — Submitted filename: PNTD-D-24-01759.docx [file pntd.0013262.s011.docx]

Review for manuscript number PNTD-D-24-01759

Reviewer’s summary:

The authors performed a cross-sectional study on 210 individuals in Venezuela on the effects of TLR9 single nucleotide polymorphism and antibody responses against different variants of Plasmodium vivax. Although the study was clear to show that there was association in specific genotypes to the antibody responses, it did not show concrete experimental evidence of the cause of differential antibody response, nor the consequence on clinical phenotype. There are some major concerns in the study that the authors should address before the manuscript can be considered for publications in PNTD.

**Major:**

1) The associations of IgG response are with symptoms and not parasitemia. Have the authors looked at the potential drivers of malaria disease, and its association with IgG?
2) The antibodies are quantified by malaria antigens. The function of the antibodies (quality), through an assay which can measure neutralization capacity, should be performed to investigate the difference between different genotype groups.
3) Did the authors validate the TLR9 sequence of the 210 participants for the 3 SNPs through Sanger sequencing? This is important and should be performed.
4) Were viral co-infections during the time of malaria infection considered for recruited individuals with ongoing malaria infections?
5) Figure 2: Can a Malaria-naïve group be added as a control to show the sensitivity and specificity of assay measuring the IgG levels against the three PvCSP variants and PvMSP-1_19_?
6) It will be great if the allele frequencies of the TLR9 gene SNPs for the general population is discussed and compared with the recruited cohort in this study. Is it in Hardy Weinberg equilibrium?
7) Are there other diseases that the TLR9 SNPs also associated with?

**Minor:**

1) Table 1: Education level seems redundant to the study.
2) Figure 1: Figure legend seems incomplete. More description on the selection or the participant inclusion criteria can be given.
3) Figure 3: Can this be converted to a dot plot, and n number be stated in the figure legend?
